# Development and Validation of a Clinical Script for Medicine Dispensing

**DOI:** 10.1111/jep.70642

**Published:** 2026-10-07

**Authors:** Sabrina Oliveira Campos de França, Audinei de Sousa Moura, Noêmia Urruth Leão Tavares, Tiago Marques dos Reis, Débora Santos Lula Barros, Rodrigo Fonseca Lima, Rafael Santos Santana

**Affiliations:** ^1^ Laboratory of Evidence and Pharmaceutical Studies (Lefar), Department of Pharmacy Faculty of Health (FS), University of Brasília (UnB) Brasília; ^2^ Federal University of Alfenas–MG (UNIFAL) Alfenas Brazil

**Keywords:** clinical practice, clinical protocols, Delphi technique, pharmaceutical dispensing, validation study

## Abstract

**Rationale:**

Pharmaceutical dispensing constitutes a fundamental clinical activity for promoting the rational use of medicines and patient safety. However, the lack of structured and validated instruments can contribute to non‐standardised practices, impacting the quality of care and the effectiveness of the interventions performed.

**Aims:**

To develop and validate the content of a clinical guide to support pharmaceutical dispensing practice.

**Methods:**

A two‐stage methodological study. Initially, the structure of the guide was draughted based on a dispensing algorithm described in the literature and adapted through a focus group comprising 14 professionals, including pharmacists and expert researchers. In the second stage, the content was validated using the Delphi Technique. The items were evaluated for representativeness, clarity, objectivity and precision, and the Content Validity Index (CVI) was calculated.

**Results:**

In the first round, most items presented a satisfactory CVI, with point adjustments needed in two sections of the guide. The main suggestions involved greater organization into a checklist format and language simplification to favour practical applicability. After the adjustments, the second round achieved full consensus among the experts, confirming the instrument's validity.

**Discussion:**

The participatory construction process and the Delphi Technique contributed to the refinement of the guide. Incorporating the experts' suggestions reinforced the importance of usability and standardization as key elements for dispensing support tools. The instrument shows potential to qualify care, reduce practice variability, and support the pharmacist's clinical reasoning. The guide demonstrated content validity and consensus among experts, proving to be a structured tool applicable to pharmaceutical dispensing practice.

## 1 Introduction

Pharmaceutical dispensing is essential for promoting the rational use of medicines, therapeutic adherence, and patient safety [1]. Given a scenario where the inappropriate use of medicines represents a public health problem with direct impacts on patient safety, qualifying this service becomes necessary for pharmaceutical services across different healthcare contexts [2, 3]. When performed in a structured manner, dispensing improves chronic disease control, reduces avoidable hospitalizations, generates cost savings for healthcare systems, and increases patient satisfaction, positively impacting clinical, humanistic, and economic outcomes [4, 5, 6, 7].

However, clinical practice presents high heterogeneity and inconsistencies, with insufficient guidance due to structural and professional limitations [3, 8, 9] The lack of practical, structured, and validated tools to support the professional favours non‐standardised practices, compromising the quality of care [10, 11, 12].

In this scenario, Rocha et al. developed a dispensing algorithm organised into clinical reasoning stages, with defined conducts for each situation identified during the service [13]. Although algorithms organise the clinical reasoning of dispensing, their application in routine services requires tools that operationalise this process in a structured and sequential manner. Using this algorithm as a baseline, the present study developed a clinical protocol to systematise the technical‐clinical content at each stage of the service.

Structured models are fundamental to qualify care and support professional decision‐making [14, 15]. To be effective, they must be developed through participatory approaches and validated by experts, ensuring clarity, objectivity and applicability [16]. Therefore, this study aimed to develop and validate a clinical protocol for medicine dispensing through participatory construction and content validation using the Delphi Technique.

## 2 Methods

This is a methodological, descriptive and exploratory study with a mixed‐method approach (qualitative and quantitative), developed in two stages: (I) participatory development of a clinical guide for pharmaceutical dispensing; (II) content validation of the guide using the Delphi Technique.

### 2.1 Stage I: Development of the Script Model

#### 2.1.1 Theoretical Construction and Reference Algorithm

The clinical structure was based on the dispensing algorithm by Rocha et al. [13], Clinical Protocols and Therapeutic Guidelines, and institutional documents. For practical adaptation, a 4‐h focus group was conducted in September 2024, comprising 14 professionals (9 pharmacists and 5 researchers).

The heterogeneity of the group aimed to integrate different perspectives [17, 18]. The discussion followed a structured guide with reference models, and data were collected through notes and collective consensus [19, 20].

#### 2.1.2 Content Structuring

The content of the clinical protocol was categorised according to the GRADE (Grading of Recommendations Assessment, Development and Evaluation) system [21], and organised into three stages:

- Initial Assessment: Guides the regulatory verification of the medicine and the technical/legal analysis of the prescription. It includes confirmation of the indication (approved or off‐label) via reference databases (Micromedex, Dynamed, UpToDate), cost/access verification and the identification of Look‐Alike Sound‐Alike (LASA) medicines to prevent errors [22]
- Guidance and Care Plan: Presents therapeutic indications classified according to the GRADE system [21], covering effectiveness, method of use and storage. Safety aspects include adverse reactions and drug interactions classified by severity level, as well as contraindications [23] The care plan is delivered verbally and tailored to the individual.
- Post‐Dispensing and Monitoring: In an adaptable checklist format [24], it covers treatment adherence guidelines and monitoring of the patient's clinical condition. Documentation is digital and includes patient identification, the dispensed medicine, interventions performed and the follow‐up plan, serving as an instrument for continuity of care and interprofessional communication.

### 2.2 Stage II: Validation of the Clinical Dispensing Protocol

#### 2.2.1 Selection of the Expert Panel

The selection of experts was carried out through purposive sampling from a contact network linked to a research group at the University of Brasília (UnB).

Inclusion criteria required practical experience in dispensing or an academic background in clinical pharmacy/pharmaceutical care. Professionals without practical experience were excluded [25]. To avoid bias, the researchers directly involved in draughting the protocol were not included in the expert panel. Initial contact was made via electronic invitation. Upon acceptance, the experts received an electronic form containing an initial section for sociodemographic and professional characterization, followed by the clinical protocol evaluation instrument.

### 2.3 Data Collection

Data collection was performed using an electronic form developed on the Google Forms platform. The instrument contemplated a dual evaluation of each section of the protocol: initially, regarding its general utility for dispensing practice (scale from useful to not useful), and subsequently, regarding content validation criteria, measured by a four‐point Likert scale.

The adopted criteria were: representativeness (the item's capacity to cover relevant aspects of clinical practice in dispensing), clarity (ease of understanding, without ambiguities), objectivity (focus on essential information, avoiding redundancies) and precision (accuracy and specificity of information, so as to minimise risks in practical application) [16]. On the Likert scale, the value 1 corresponds to ‘criterion not met’ and the value 4 to ‘criterion fully met’.

For evaluation purposes, the form was accompanied by an exemplary model of a clinical protocol draughted for the dispensing of medicines used in HIV pre‐ and post‐exposure prophylaxis (PrEP/PEP), with the objective of contextualising the practical application of the instrument. The suggestions received were analysed for their relevance and applicability to the generic clinical protocol under development.

In addition to the quantitative evaluation, open fields were provided for qualitative comments, enabling the experts to record observations and suggestions for adjustments to the protocol items.

### 2.4 Application of the Delphi Technique

Validation was conducted using the Delphi Technique in successive rounds to reach consensus among the participants [26, 27]. The first round took place in person during a meeting dedicated to the presentation and discussion of the initial protocol model. At this stage, the experts analysed the criteria of representativeness, clarity, objectivity and precision.

The quantitative analysis was based on the Content Validity Index (CVI), an indicator that expresses the proportion of agreement among experts regarding the adequacy of each item [16]. The CVI was calculated as the ratio between the number of positive responses (values 3 or 4 on the Likert scale) and the total number of responses obtained for each item, according to the formula:

$$\mathrm{CVI}=\frac{\text{number}\;\text{of}\;3\;\text{or}\;4\;\text{responses}}{\text{total}\;\text{number}\;\text{of}\;\text{responses}}$$

Items that achieved a CVI equal to or greater than 0.80 were considered valid [16, 28]. Simultaneously, qualitative contributions from the open‐ended fields were grouped by similarity and selected by consensus by the research group to refine the protocol.

Items that did not reach the 0.80 consensus were revised and submitted to a second round (electronic and without open‐ended fields). This new version was sent to the same experts in a comparative format (‘before and after’) for a new quantitative evaluation.

### 2.5 Data Analysis

Quantitative data were analysed by calculating the Content Validity Index (CVI), performed manually using an electronic spreadsheet. Items that achieved a CVI equal to or greater than 0.80 were considered valid, in accordance with methodological criteria established in the literature [16, 28, 29].

To mitigate bias in the process, measures were taken such as ensuring the anonymity of responses among participants, predefining the cutoff point for consensus, and excluding the researchers involved in draughting the protocol from the expert panel.

## 3 Results

### 3.1 Development of the Clinical Dispensing Protocol

The development process resulted in the Clinical Dispensing Protocol, a digital instrument designed to support the pharmacist's clinical reasoning. Structured into three sequential stages and sub‐sections, the protocol combines text and checklist formats, making it adaptable to each patient's medicine and clinical condition. Its therapeutic indications are classified according to the GRADE system evidence levels [21]. The structure comprises:

1. Initial Assessment: Access and Availability.
2. Guidance and Care Plan: Indication, Effectiveness and Mode of Use, Safety and Special Populations.
3. Post‐Dispensing and Monitoring: Adherence and Follow‐up, and Records.

### 3.2 Validation of the Clinical Dispensing Protocol

#### 3.2.1 Characterization of the Expert Panel

The first round of the Delphi Technique included the participation of 30 experts, all pharmacists, the majority being female, with a predominance in the 31 to 40 age range. Professional experience ranged from 1 to more than 20 years, with 46.7% of the experts reporting up to 5 years of experience and 33.3% between 11 and 20 years. The second round included 17 experts, maintaining the heterogeneity of the sample—a reduction expected in this type of design, given that the literature points to significant drops in response rates between successive rounds [27, 30]. The characterization of the experts is described in Table 1.

**Table 1:** Characterization of expert participants in the Delphi Technique.

	First‐round participants (*n *= 30)		Second‐round participants (*n *= 17)	
Characteristic	%	*n*	%	*n*
Gender				
Female	66.7%	20	58.8%	10
Male	33.3%	10	41.2%	**7**
Age				
20–30 years	10%	3	0	0
31–40 years	53.3%	16	76.5%	13
41–50 years	23.3%	7	5.9%	1
51–60 years	10%	3	17.6%	3
61 years or older	3.3%	1	0	0
Professional experience time				
Up to 5 years	46.7%	14	35.3%	6
6 to 10 years	10%	3	23.5%	4
11 to 20 years	33.3%	10	29.4%	5
More than 20 years	10%	3	11.8%	2

*Source:* Own authorship.

##### 3.2.1.1 First Round of the Delphi Technique

In the first round, 30 experts evaluated the protocol across four criteria (Table 2). Most items presented a high CVI (close or equal to 1.0), indicating strong agreement [31, 32]. While representativeness, clarity and precision reached consensus in all sections, the objectivity criterion fell below the 0.80 cutoff point in the Access and Availability and Safety sections (0.70 in both), requiring structural revision.

**Table 2:** Content Validity Index by section and technical criterion: 1st round of the Delphi Technique (*n* = 30).

Section	Criteria			
	Representativeness	Clarity	Objectivity	Precision
Access and availability	0.83	0.83	**0.70** *	1.00
Indication	0.93	0.90	0.80	1.00
Mode of use and effectiveness	0.96	0.96	0.86	0.93
Safety	0.90	0.83	**0.70** *	0.93
Special populations	0.90	0.90	0.83	0.93
Records and follow‐up	0.93	1.00	0.93	0.90

*Source:* Own authorship.

* Items that did not reach consensus in the first round of the Delphi Technique (CVI < 0.80).

Qualitative feedback yielded 21 comments for Access and Availability and 15 for Safety. The research group analysed these suggestions by content similarity, incorporating relevant and applicable modifications into the protocol [33], as detailed in Table 3.

**Table 3:** Suggestions from experts and actions taken in the sections that did not reach consensus.

Section	Suggestions from experts	Action taken
Access and Availability	Transform content into a checklist format, making it more visual and objective	Restructuring of the section into an operational format with topics and a checklist
Access and Availability	Remove non‐essential information for the moment of dispensing	Elimination of content that does not directly impact the act of dispensing
Access and Availability	Distinguish the pharmacist's technical reference information from patient guidance	Reorganization of content separating reference information from user guidance
Access and Availability	Include necessary documents and conducts for specific situations	Insertion of a checklist for mandatory documents and standardised conducts
Access and Availability	Include identification of the patient's needs as a preliminary stage to the care plan	Incorporation of an initial assessment of patient needs
Safety	Cut down information and make content more objective	Review and synthesis of content focusing on essential information
Safety	Highlight the most common adverse reactions and organise them by frequency	Reorganization of adverse reactions in descending order of frequency by system
Safety	Make conducts clearer in case of adverse reactions and interactions	Inclusion of clear conducts for managing adverse reactions and interactions
Safety	Distinguish technical reference information from patient guidance	Reorganization separating technical information from user guidance

*Source:* Own authorship.

##### 3.2.1.2 Second Round of the Delphi Technique

Following the modifications, the updated protocol was presented to the same experts in a comparative format via a new electronic form. As shown in Table 4, all re‐evaluated items achieved a CVI of 1.00, indicating unanimous agreement. Since no open fields were included and no new queries arose, the validation process was concluded.

**Table 4:** CVI of the re‐evaluated criteria: 2nd round of the Delphi Technique (*n* = 17).

Section	Re‐evaluated criterion	CVI (2nd round)
Access and Availability	Objectivity	1.00
Safety	Objectivity	1.00

*Source:* Own authorship.

The experts' qualitative feedback led to a reformulated protocol focused on utility and ease of use during dispensing, with a comparison between the initial and validated models presented in Chart 1. The final structure of the Clinical Dispensing Protocol, detailing the technical‐clinical content required for any medicine or condition across each stage and sub‐section, is described in Chart 2.

**Chart 1:**
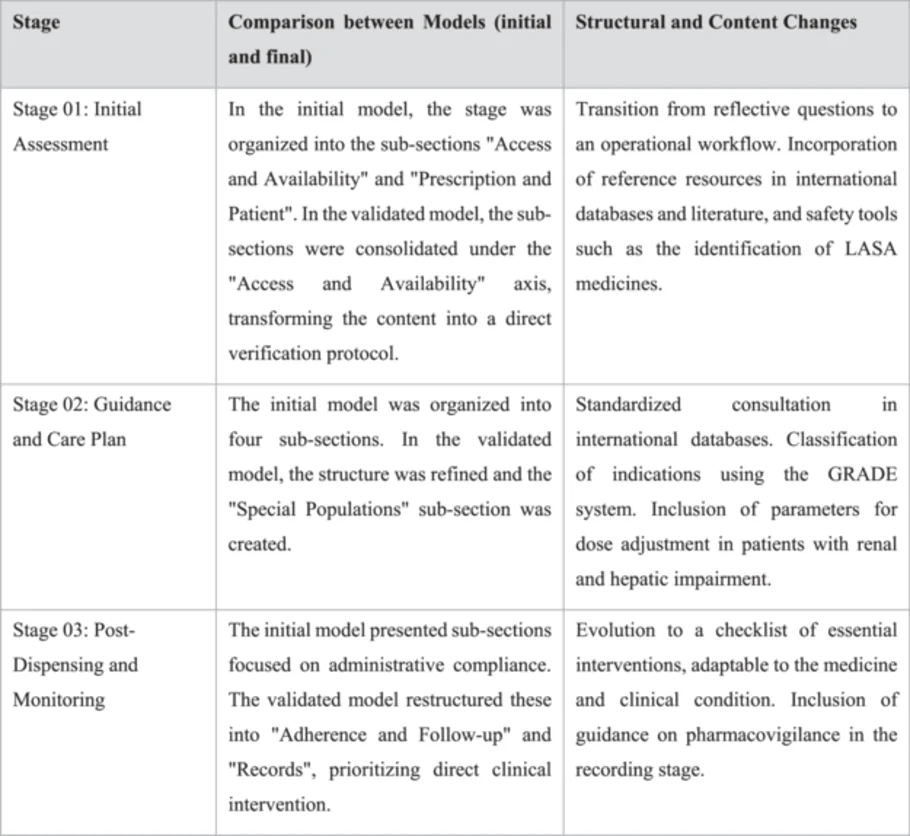
Comparison between the initial model and the validated model of the Clinical Dispensing Protocol. *Source:* Own authorship.

Chart 2Structure and content of the Clinical Dispensing Protocol. *Source:* Own authorship.

**Figure:**
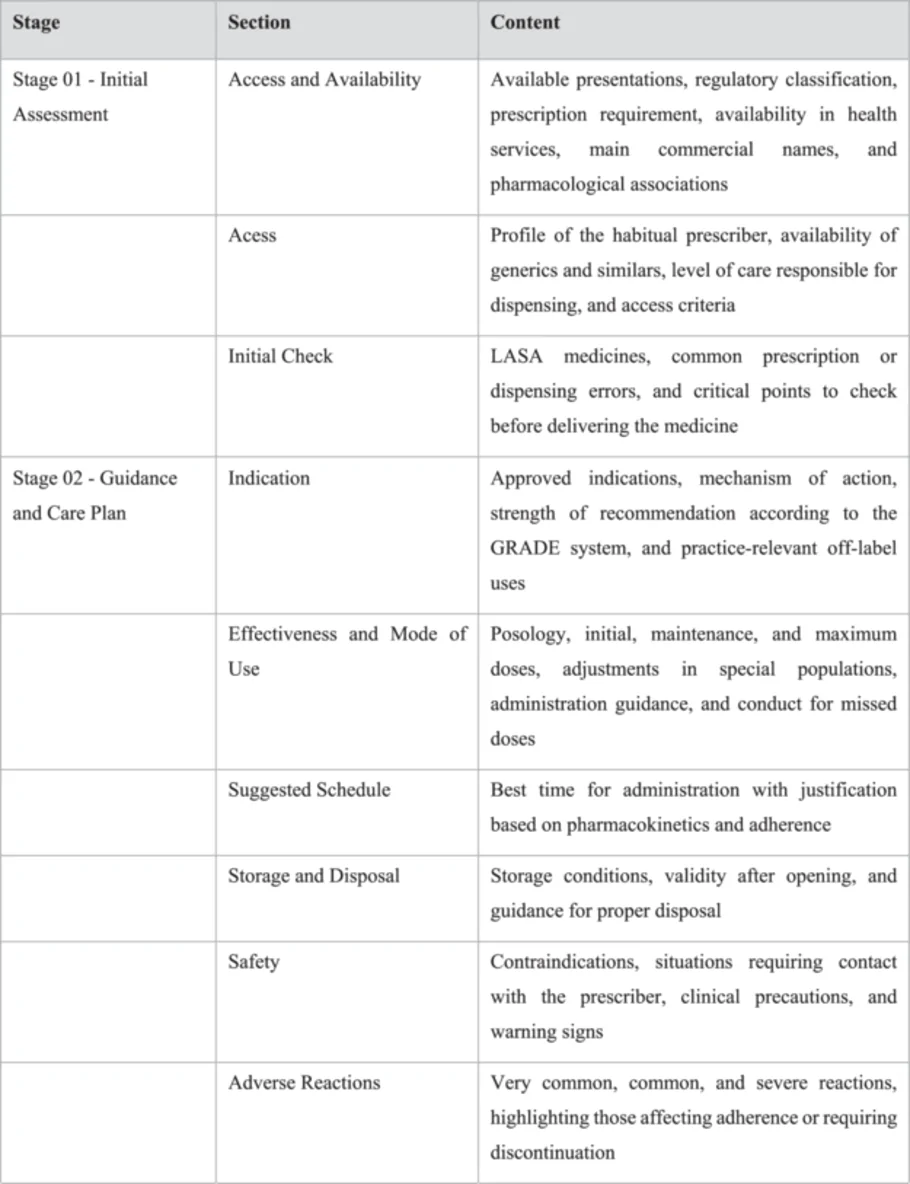

**Figure:**
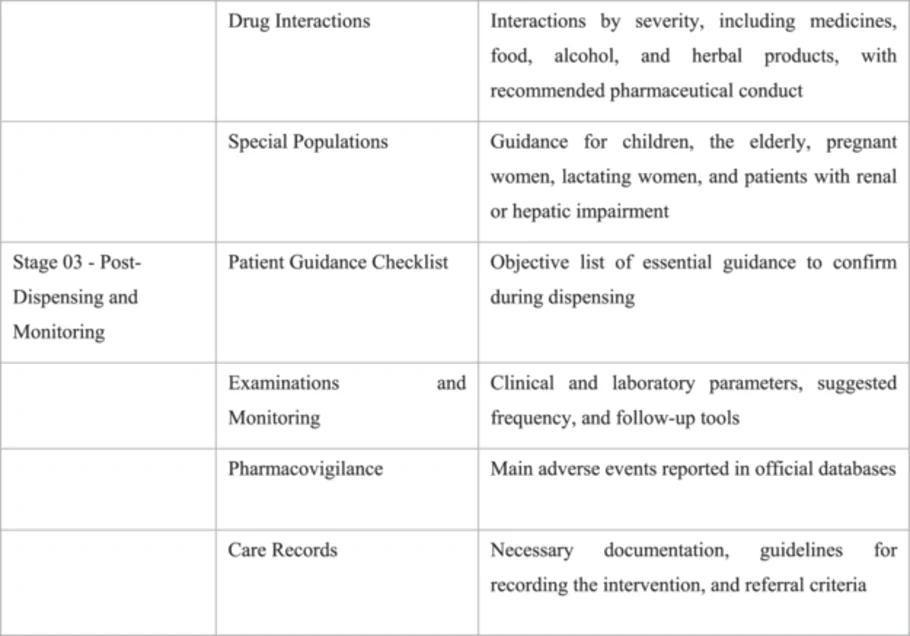

## 4 Discussion

The Clinical Dispensing Protocol is organised into three sequential stages—Initial Assessment, Guidance and Care Plan, and Post‐Dispensing and Monitoring—designed to support the pharmacist's clinical reasoning. This structured approach aligns with literature showing that systematised pharmaceutical action effectively prevents medication errors and qualifies patient care [30, 34, 35, 36]

The Initial Assessment stage focuses on the technical and legal analysis of the prescription. Systematic review by the pharmacist is one of the most effective strategies to detect and prevent frequent prescription and dispensing errors, such as inconsistencies in dosage, frequency and duration [37, 38]. This stage also includes confirming the therapeutic indication (approved or *off‐label*) via reference databases and identifying Look‐Alike Sound‐Alike (LASA) medicines, which are globally recognised sources of preventable adverse events [22].

The Guidance and Care Plan stage structures the delivery of evidence‐based information. Classifying indications via the GRADE system enhances transparency and supports clinical decision‐making [21]. Providing tailored guidance on administration, dosage, schedules, storage, adverse reactions, contraindications and drug interactions directly impacts pharmacotherapy safety and effectiveness [27, 39]. Additionally, incorporating specific recommendations for special populations addresses the need for individualised care due to distinct physiological and clinical characteristics [23].

The Post‐Dispensing and Monitoring stage focuses on therapeutic follow‐up. Structured pharmaceutical interventions to monitor treatment significantly improve adherence and clinical outcomes in chronic conditions [40, 41]. Including guidance on adherence, clinical monitoring and pharmacovigilance strengthens continuity of care and enables early identification of medicine‐related problems [42, 43]. Furthermore, digitally recording these interventions ensures interprofessional communication, traceability and patient safety [44].

Content validation via the Delphi Technique allowed progressive improvement of the protocol based on expert input, ensuring greater robustness [30, 34, 35]. The panel comprised pharmacists with extensive experience in dispensing practice and clinical pharmacy research, a recruitment method recommended to guarantee deep thematic expertise [45, 46].

In the first round, the 30 experts evaluated the protocol across four criteria. Representativeness, clarity, and precision reached consensus in all sections, with CVIs close or equal to 1.0, well above the literature's recommended 0.78 threshold. Only the objectivity criterion fell below the cutoff in the Access and Availability and Safety sections, signalling the need for structural revision. Incorporating the experts' qualitative suggestions regarding wording and organization successfully enhanced the instrument's clarity and objectivity [33].

In the second round, all re‐evaluated items achieved a CVI of 1.00. The drop from 30 to 17 experts between rounds is expected in Delphi designs, as the literature notes significant response rate declines across successive stages [31]. Unlike studies requiring three or more rounds [47, 48], two were sufficient here, likely due to the high quality of the qualitative feedback and the clarity of the subsequent modifications.

The development and validation of this Clinical Dispensing Protocol offer pharmacists a tool to identify patient needs and design individualised care plans, qualifying clinical practice [49]. The literature highlights that structured instruments are essential to organise care by establishing clear, standardised recommendations that guide professional interventions [33, 49].

Although widely used, the Delphi Technique presents limitations, such as potential biases in expert selection and methodological heterogeneity across studies [27, 30]. To mitigate these risks, this study excluded the protocol's developers from the panel, guaranteed response anonymity, and predefined the consensus cutoff point. Nevertheless, the reduction in participants between rounds may have influenced final agreement levels [28, 30]. Furthermore, this study was restricted to content validation and did not evaluate clinical effectiveness in real practice.

Future research should assess the protocol's use and acceptability across diverse healthcare settings (primary care, hospitals, pharmacies) and measure its impact on clinical outcomes, including adherence, patient satisfaction and adverse events.

## Ethics Statement

Approved by the Research Ethics Committee of the Faculty of Health Sciences of the University of Brasília (CEP/FS‐UnB) under CAAE 77973224.6.0000.0030 (Opinion No. 6,817,982), the study formalised expert participation via an electronic Informed Consent Form (ICF). Individual responses were kept confidential to ensure evaluation independence and minimise peer influence.

## Conflicts of Interest

The authors declare no conflicts of interest.

## Data Availability

The data that support the findings of this study are available on request from the corresponding author. The data are not publicly available due to privacy or ethical restrictions.
